# Genome wide association study identifies *KCNMA1 *contributing to human obesity

**DOI:** 10.1186/1755-8794-4-51

**Published:** 2011-06-28

**Authors:** Hong Jiao, Peter Arner, Johan Hoffstedt, David Brodin, Beatrice Dubern, Sébastien Czernichow, Ferdinand van't Hooft, Tomas Axelsson, Oluf Pedersen, Torben Hansen, Thorkild IA Sørensen, Johannes Hebebrand, Juha Kere, Karin Dahlman-Wright, Anders Hamsten, Karine Clement, Ingrid Dahlman

**Affiliations:** 1 Department of Biosciences and Nutrition, Karolinska Institutet, SE-141 83 Huddinge, Sweden; 2 Clinical Research Centre, Karolinska University Hospital, SE-141 57 Stockholm, Sweden; 3 Department of Medicine at Karolinska Institutet and Karolinska University Hospital, SE-141 86 Stockholm, Sweden; 4 INSERM, U-557/INRA U-1125, CNAM, UP13, CRNH-IdF, 93017 Bobigny, France; University Paris 13, 93017, Bobigny, France; AP-HP, Avicenne Hospital, 93017 Bobigny, France; 5 Cardiovascular Genetics Group, Atherosclerosis Research Unit, Department of Medicine Solna, Karolinska Institutet, SE-17176 Stockholm, Sweden; 6 Department of Medical Sciences, Molecular Medicine, Science for Life Laboratory, Uppsala University, Uppsala, Sweden; 7 Hagedorn Research Institute, Gentofte,, Copenhagen, Denmark; 8 Center of Basic Metabolic Research, Faculty of Health Sciences, University of Copenhagen, Denmark; 9 Institute for Preventive Medicine, Copenhagen University Hospital, Center for Health and Society, Copenhagen, Denmark; 10 Department of Child and Adolescent Psychiatry of the University of Duisburg-Essen, Essen, Germany; 11 INSERM, U-872, Nutriomique (team 7) 75006 Paris, France; University Pierre and Marie Curie-Paris 6, Cordeliers Research Center, 75006 Paris, France; AP-HP, Pitié-Salpétrière Hospital, 75013 Paris, France

## Abstract

**Background:**

Recent genome-wide association (GWA) analyses have identified common single nucleotide polymorphisms (SNPs) that are associated with obesity. However, the reported genetic variation in obesity explains only a minor fraction of the total genetic variation expected to be present in the population. Thus many genetic variants controlling obesity remain to be identified. The aim of this study was to use GWA followed by multiple stepwise validations to identify additional genes associated with obesity.

**Methods:**

We performed a GWA analysis in 164 morbidly obese subjects (BMI:body mass index > 40 kg/m^2^) and 163 Swedish subjects (> 45 years) who had always been lean. The 700 SNPs displaying the strongest association with obesity in the GWA were analyzed in a second cohort comprising 460 morbidly obese subjects and 247 consistently lean Swedish adults. 23 SNPs remained significantly associated with obesity (nominal *P*< 0.05) and were in a step-wise manner followed up in five additional cohorts from Sweden, France, and Germany together comprising 4214 obese and 5417 lean or population-based control individuals. Three samples, n = 4133, were used to investigate the population-based associations with BMI. Gene expression in abdominal subcutaneous adipose tissue in relation to obesity was investigated for14 adults.

**Results:**

Potassium channel, calcium activated, large conductance, subfamily M, alpha member *(KCNMA1) *rs2116830*G and *BDNF *rs988712*G were associated with obesity in five of six investigated case-control cohorts. In meta-analysis of 4838 obese and 5827 control subjects we obtained genome-wide significant allelic association with obesity for *KCNMA1 *rs2116830*G with *P *= 2.82 × 10^-10 ^and an odds ratio (OR) based on cases vs controls of 1.26 [95% C.I. 1.12-1.41] and for *BDNF *rs988712*G with *P *= 5.2 × 10^-17^and an OR of 1.36 [95% C.I. 1.20-1.55]. *KCNMA1 *rs2116830*G was not associated with BMI in the population-based samples. Adipose tissue (*P *= 0.0001) and fat cell (*P *= 0.04) expression of *KCNMA1 *was increased in obesity.

**Conclusions:**

We have identified *KCNMA1 *as a new susceptibility locus for obesity, and confirmed the association of the *BDNF *locus at the genome-wide significant level.

## Background

Several susceptibility loci for high BMI and obesity have recently been identified by genome-wide association (GWA) analyses of both large population-based samples and samples including extreme obese phenotypes [1-11]. Analysis of the latter group is based on the assumption that subjects with an extreme phenotype, such as morbid obesity, may be enriched for variants that influence the risk of developing obesity [7]. It is possible that extreme phenotypes of common disorders are due to rare. but highly penetrant, alleles that are difficult to identify by GWA analysis based on common single nucleotide polymorphisms (SNPs). However, based on GWA analysis of extreme obesity, Cotsapas et al found no evidence for rare highly penetrant alleles and concluded that most cases of severe obesity are extremes of the phenotypic spectrum rather than a distinct condition [6]. However, this does not exclude the existence of rare variants underlying specific cases of morbid obesity [12-14].

Susceptibility genes for human obesity are believed to act primarily on the central regulation of food intake. Three reported susceptibility loci for obesity harbour genes that are known to be involved in catabolic hypothalamic pathways (*MC4R, PCSK1*, and *BDNF*) and many others contain genes that are highly expressed in the central nervous system [11,15,16]. However, studies in mice mutated for *Fto*, the homolog of the human obesity susceptibility gene *FTO*, have demonstrated peripheral metabolic effects of this gene [17,18]. Overall, the mechanisms of action of most obesity genes are not well understood and adipose tissue, as well as skeletal muscle, which are sites for storage, release, and metabolism of fatty acids, may be involved.

Overall, the reported genetic variation in obesity explains only a minor fraction of the total genetic variation expected to be present in the population. In the most recent GWA meta-analysis, 32 obesity loci explained together only about 2-4% of the genetic variation in BMI [11]. Thus, much of the genetic variation controlling obesity remains to be discovered [11,15,19]. In this study we report the identification of a novel susceptibility gene for obesity by GWA analysis of morbidly obese cases and controls that have a lifelong history of leanness. The association for this gene was confirmed in five independent case *versus *control cohorts, as well as in population based samples. Finally, we quantified the expression of the novel gene contributing to obesity in human adipose tissue samples.

## Methods

### Cohorts

The cohorts for genetic studies of obesity and BMI are described in Tables 1 and 2. The GWA analysis was performed for the Swedish cohort 1 comprising subjects (age 16-73 years) with morbid obesity (BMI ≥40.0 kg/m^2^), and lean subjects > 45 years old who never had been overweight (BMI always < 25.0 kg/m^2^). All subjects in Cohort 1 were at least third generation Scandinavian and lived in Sweden. Swedish cohort 2 was used for initial replication of the GWA results and had identical inclusion criteria. Swedish cohort 3 comprised obese adults with BMI ≥30.0 kg/m^2 ^and lean subjects who were > 25 years old and had BMI < 25.0 kg/m^2^, all having European ancestry and from the greater Stockholm area. Cohorts 1, 2 and 3 were selected according to the above BMI inclusion criteria amongst subjects recruited by local advertisements or amongst participants in population-based surveys or case-control studies of myocardial infarction (282 had myocardial infarction, of which 89 were obese). These cohorts included subjects diagnosed with type 2 diabetes (n = 301), hypertension (n = 810) or dyslipidemia (n = 385). Patients with chronic inflammatory diseases other than cardiovascular disease, type 1 diabetes mellitus, renal insufficiency (serum creatinine > 200 μmol/L), drug addiction or psychiatric disease were excluded.

**Table 1:** Cohorts for genetic studies

				obese cases			lean and population-based controls		
	Cohort	**SNP**§	Nationality	female/male	BMI* **(kg/m^2^)**	age* **(years)**	female/male	BMI* **(kg/m^2^)**	age* **(years)**
Discovery	**1**	406,177	Swedish	131/33^§^	44.7 ± 4.7	43.8 ± 12.6	137/26	22.2 ± 1.8	51.6 ± 6.1
Replication	**2**	700	Swedish	370/90^§§^	44.6 ± 4.6	42.2 ± 13.2	206/41	22.6 ± 1.7	51.6 ± 5.3
Replication	**3**	21	Swedish	1025/789	37.1 ± 5.4	46.6 ± 11.4	819/885	22.8 ± 1.7	48.7 ± 10.8
Replication	4**	21	French	641/344	33.7 ± 8.2	14.1 ± 5.0	289/243^#^	18.7 ± 3.3	11.8 ± 1.6
Replication	**5**	2	French	682/246	48.5 ± 7.6	43.0 ± 12.1	1630/1108^#^	23.8 ± 3.5	49.7 ± 6.3
Replication	6**	2	German	278/209	33.4 ± 6.8	14.4 ± 3.7	271/171	18.3 ± 1.1	16.1 ± 5.8
Total				3127/1711			3353/2474		

* Values are mean ± SD; ** Cohorts comprising children in which case BMI Z-scores were used to define obesity status as defined in Methods.

§ Number of SNPs genotyped in each cohort. The 700 most significantly obesity associated SNPs in the GWA were analyzed in cohort 2 (threshold *P *= 0.003). Subsequently, nominal association with obesity was used as threshold to determine whether a SNP would be followed up by genotyping additional cohorts. # population-based controls

**Table 2:** Population-based cohorts for analysis of BMI

nationality	cohort	Female/male	BMI* (kg/m^2^)	age* (years)
French	5§	1630/1108	23.8 ± 3.5	49.7 ± 6.3
Swedes	7	545/0	25.3 ± 3.9	43.9 ± 4.3
Danes	8	0/850	26.1 ± 3.6	43.0 ± 6.0

Values are mean ± SD; **Number of SNPs that were genotyped in each cohort.

§ This cohort is identical to the population-based controls in table 1

Cohort 4 comprised French obese and population-based control children. Obese children living in the Paris area were consecutively recruited starting in 2001. Obesity was assessed by BMI Z-scores [standard deviation (SD) over mean BMI at a given age and sex for a French reference population]. Obese children had a BMI Z-score ≥3 SD above the means specific for age and sex in normal French children as described previously [20,21]. The control children participated in a population-based physical activity study [22]. Phenotypes were collected before the physical activity intervention.

Cohort 5 comprised adult French obese cases and population-based control subjects. Obese adults living in the Paris area were consecutively recruited and had morbid obesity (BMI ≥40.0 kg/m^2^). The adults in the control group were participants of SU.VI.MAX, which is a study to test antioxidant supplementation [23]. A subset of 2738 subjects living in Paris was used as controls in the present study. Phenotypes were collected at study entry. Women are more likely to respond to advertisements for participation in obesity research, which explains the female-biased gender ratio in cohorts 1 and 2, and among the obese in cohorts 4 and 5.

Cohort 6 encompassed German extremely obese children and adolescents (mean BMI Z score 4.6 ± 2.3) and adult lean controls (mean BMI Z score: -1.4 ± 0.4) (for details see [3]). The BMI of the obese patients was above the 90th BMI percentile for German children and adolescents (see http://www.mybmi.de). 91% of the obese patients had a BMI above the 97th percentile. The lean adult controls were all students at Marburg University.

In order to assess the association between SNPs and BMI, two additional population-based adult cohorts were genotyped, besides the controls in cohort 4 (Table 2). Cohort 7 encompassed 545 adult women who were recruited in Stockholm (see [24] for details). Cohort 8 encompassed 850 Danish men randomly selected from the mandatory draft board examinations 1943-1977 and investigated 1998-2000 (see [25] for details). Thus, 2175 women (mean age 47 years) and 1958 men (mean age 50 years) were genotyped in the population-based survey. The population based cohorts 7 and 8 contained very few subjects with morbid obesity and were therefore not included in the meta-analysis of obesity in cases versus controls. Case-control and population-based samples have apparently no familial links although relatedness has not been firmly tested. However, the absence of apparent relatedness in cohort 1 was supported by the strong overlap between expected and observed p-values in the Q-Q plot (additional file 1, Figure S1).

Subjects included in analysis of human abdominal subcutaneous adipose tissue were from cohort 3 (see above). In these studies obesity was defined as BMI > 30 kg/m^2 ^and leanness as BMI < 25 kg/m^2^. All subjects were healthy according to self-report. Expression of specific genes in relation to obesity was investigated in seven lean (5 women and 2 men with BMI 23.3 ± 1.7 kg/m^2 ^and age 33.0 ± 9.8 years) and seven obese subjects (6 women and 1 man with BMI 34.4 ± 5.9 kg/m^2 ^and age 48.6 ± 12.2).

### Ethical approval

The studies had been approved by the local Ethics Committee. Each subject gave informed written consent to the study. For subjects less than 18 years of age, authorization was obtained from the parents.

### Genotyping

**Figure 1:**
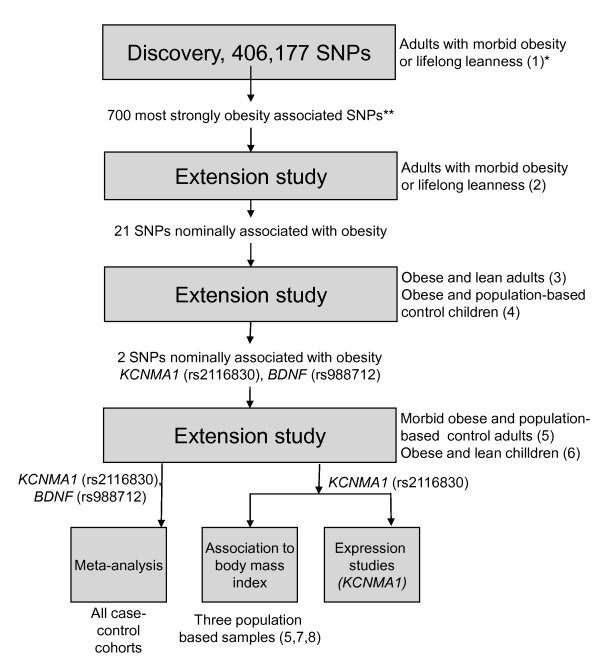
**Strategy for Genome wide association analysis**. *Phenotypes of studied cohorts with cohort number within parenthesis. **Number of SNPs taken forward for genotyping and analysis in subsequent cohorts.

The strategy used to find obesity genes is shown in Figure 1. The GWA study was performed for Cohort 1 on the Affymetrix 500K Gene chip arrays (Affymetrix, Santa Clara, CA) assaying 489,922 autosomal SNPs. Genotypes were first called using the DM model implemented in GeneChip^® ^Genotyping Analysis Software (GTYPE) Version 4.0 (Affymetrix) with default parameters. All arrays had call rates ≥93% and were then assessed using the BRLMM model. The average call rate based on the BRLMM model was 98.6%. Quality control of SNPs was performed as described in the additional file 1. After quality control, a total of 406,177 autosomal SNPs were analyzed for association with obesity. The additional file 1, Table S1, shows the result of the quality control and chromosomal distribution of analyzed SNPs.

The statistical power of cohort 1 was limited and we did not expect to obtain genome-wide significance in this discovery cohort. We therefore followed up a large number of nominally significant obesity-associated SNPs in a step-wise manner by genotyping additional cohorts, starting with cohort Swedish 2. We selected the 755 SNPs with the lowest p-values for allelic association with obesity in the GWA study, with the exception that for SNPs in close linkage disequilibrium according to our GWA data (SNPs which were carried on identical haplotypes according to HaploView with default settings) only one was included for further analysis, for genotyping using Illumina Golden Gate assays (Illumina Inc.). Thus, the 755 SNPs represented unlinked loci across the human genome. 755 SNPs were chosen since this number would maximize the efficient use of the genotyping platform. As a consequence of the applied selection method the threshold for following up and genotyping a SNP in cohort 2 was a p-value of 0.003 (p-value range 0.003 to 1.17 × 10^-6^) for association with obesity in the GWA. Of the SNPs selected for follow-up, 700 were successfully genotyped.

Subsequent genotyping for cohorts 3, 4, and 5 (cases), and 7 were performed with matrix-assisted laser desorption/ionization time-of-flight mass spectrometry (SEQUENOM). The SU.VI.MAX control cohort 5 and the cohort 8 were genotyped by TaqMan (Applied Biosystems, Foster City, CA and K Biosciences, Herts, UK, respectively). Thus all above cohorts were de novo genotyped in this project. By contrast for cohort 7 we performed *in silico *replication using available Affymetrix 6.0 GWA genotypes [3]. All of cohort 1 and cohort 2 were genotyped twice for rs2116830 with different methods and all genotypes were concordant between platforms.

### Adipose tissue samples

Abdominal subcutaneous biopsies were obtained during elective surgery for non-malignant disorders after an overnight fast. Fat cells were isolated as described [26]. Adipose tissue pieces (300 mg) or 200 μl isolated adipocytes were immediately frozen in liquid nitrogen and kept at -70°C for subsequent RNA isolation.

### cDNA synthesis and quantitative real-time PCR

Total RNA was extracted from adipose tissue samples and transcribed to cDNA as described previously [27]. *KCNMA1 *and the reference gene *18S *were quantified using SYBR Green-based quantitative real-time PCR (qRT-PCR). Primers were for *KCNMA1: *5'- CAGAAGTTGGCTTGGTTTGAG -3' and 5'- ATGGAGAGCAGATTCACCAG -3', and for 18S: 5'- CACATGGCCTCCAAGGAGTAAG -3' and 5'- CCAGCAGTGAGGGTCTCTCT -3'. 5 ng of cDNA was mixed with gene specific primers (final concentration 300 nM) and IQ™ SYBR Green supermix (Bio-Rad Laboratories, Hercules, CA, USA) and amplified with an iCycler IQ (Bio-Rad Laboratories, Hercules, CA, USA) according to the manufacturer's instructions. Dissociation curve analyses and agarose gel electrophoresis were used to validate that a single amplicon was amplified. All reactions were run in duplicate. Relative gene expression was calculated using a direct comparative method (User Bulletin #2, Applied Biosystems).

### Statistical analysis

Hardy-Weinberg equilibrium (HWE) of the genotypic frequencies among cases and controls were tested separately prior to association analyses. *P*< 0.001 in controls was used as cut-off for exclusion of failed SNPs from further analysis. This is a conventional cut-off for exclusion of failed SNPs in GWA studies.

Association between single SNPs and obesity status was performed in the same manner in the GWA study and in the extension studies. Initially association was evaluated by allelic association; allele frequencies were compared between obese and control populations by the χ^2 ^test using in-house programs. We chose a test of allelic association since we had no a priori assumption which genetic model best explains SNP association with obesity. Distribution of p-values in the GWA study was presented as Q-Q and Manhattan plots, which were generated using the R package [28]. We performed meta-analysis of the association between obesity and individual SNPs across the six investigated case-control cohorts. The inverse variance method was used for pooling of cohort results. The combination of data and the combined value of the odds ratio (OR) and 95% confidence interval (C.I.) were calculated using the random effects estimate method implemented in the R package, and meta-analysis plots were generated using the R package. For the genetic loci that displayed genome-wide allelic association with obesity, model-based tests were carried out to evaluate association of genotype with obesity using logistic regression implemented in PLINK [29]. Models were tested for the effect allele [30].

rs2116830 was evaluated for association with BMI in population-based cohorts by linear regression as implemented in PLINK. The Wald test was performed to give asymptotic p-value for significance. Statistical power was calculated as described [31] based on mean and SD for BMI of all adult populations-based samples.

Differences in *KCNMA1 *mRNA between two groups were evaluated by two-sided unpaired Student's t test. Values are mean ± SD.

## Results

We performed a GWA analysis of morbidly obese cases and controls that have a lifelong history of leanness. The distribution of p-values for all SNPs that were analyzed for association with obesity is shown in the additional file 1, Figure S1 (Q-Q plot) and S2 (Manhattan plot). No SNP displayed genome-wide significant association with obesity in the GWA, that is *P*< 10^-7^. The SNPs showing the strongest association in the GWA were analyzed in additional case-control cohorts 700 SNPs were successfully genotyped in cohort 2. Of these, 23 were nominally associated with obesity with consistent effect direction as observed for the GWA (additional file 1, Table S2). 21 of these SNPs (one SNP assay failed and another SNP had already been investigated as a candidate gene [32]) were subsequently analyzed in Swedish cohort 3 and French cohort 4. Two of the 21 SNPs displayed consistent effect direction and nominal significant association with obesity in these cohorts, rs2116830, and rs988712 (additional file 1, Table S2, Table 3). These two SNPs were subject to further confirmation.

**Table 3:** GWA SNPs with confirmed allelic association with obesity in extension studies

Chr.	Gene	Effect allele*	Cohort**	Call rate (%)	Obese		Controls		*P*§§
					**GG/GT/TT**	**%§**	**GG/GT/TT**	**%§**	

10	*KCNMA1*	rs2116830G	1	98	121/35/4	87	91/59/9	76	5.0 × 10^-04^
			2	99	314/126/9	84	153/74/13	79	0.019
			3	99	1232/499/53	83	1108/522/61	81	0.018
			4	98	684/232/25	85	348/161/16	82	0.024
			5	97	598/263/26	82	1065/576/81	79	0.0017
			6	100	318/125/9	83	295/128/12	84	0.35
			Pooled		3267/1180/126		3060/1520/192		2.8 × 10^-10^
11	*BDNF*	rs988712G	1	91	104/53/4	81	86/67/10	73	0.0017
			2	99	300/140/13	82	139/83/16	76	0.0095
			3	99	1126/590/66	80	976/595/112	76	3.9E-05
			4	98	609/283/34	81	273/199/50	71	2.2 ×10^-9^
			5	95	540/290/41	79	942/642/106	75	0.0019
			6	99	273/148/25	78	246/151/33	75	0.13
			Pooled		2952/1504/183		2662/1737/327		5.2 × 10^-17^

* All SNPs are in HWE with *P*> 0.05. ** Details about cohorts are given in table 1. § Frequency of effect allele among obese cases and controls. §§ Allele frequencies were compared by Chi^2^-test.

**Figure 2:**
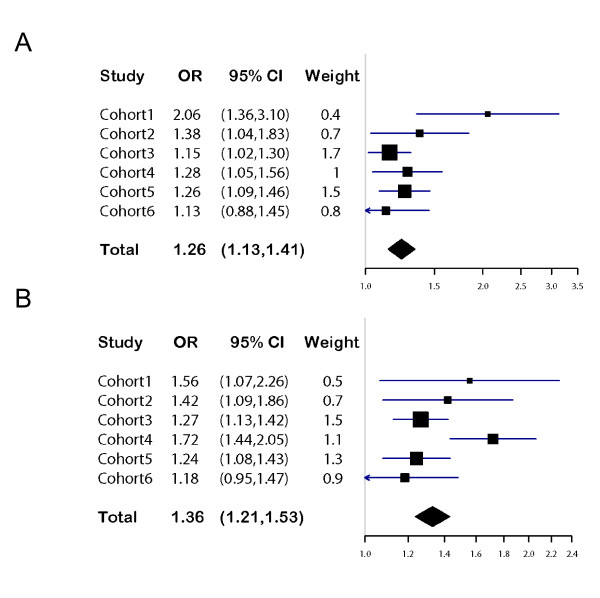
Meta-analysis of association between (A) *KCNMA1 *rs2116830*G, and (B) *BDNF *rs988712*G with obesity in six cohorts. For meta-analysis results were pooled using the inverse variance method. Combined OR and C.I. were calculated using the random effects estimate method implemented in R.

The G-allele of rs2116830, located in intron 28 of the gene Potassium channel, calcium activated, large conductance, subfamily M, alpha member *(KCNMA1) *on chromosome 10, showed consistent effect direction and significant allelic association with obesity in the French cohort 5 comprising morbidly obese adults and population-based adult control subjects (*P *= 0.0017), but not in the German cohort 6 comprising morbidly obese youth and adult lean controls (P = 0.35) (Table 3, Figure 2A). The overall meta-analysis p-value for cases vs controls showed genome-wide significance (*P *= 2.82 × 10^-10^) with an odds ratio (OR) of 1.26 [95% C.I. 1.12-1.41], with no statistical evidence for heterogeneity in impact on obesity between cohorts (Figure 2A). The impact of the G-allele of rs2116830 on obesity under different genetic models was tested in a joint analysis of all case-control cohorts. The recessive, *P *= 2.5 × 10 ^-9 ^(OR 1.30 [95% C.I. 1.19-1.42]), but not the additive or dominant genetic model reached genome-wide significance (Table 4).

**Table 4:** Association of SNPs with obesity under different genetic models

SNP	Gender	Genetic model	Obese (n)	Control (n)	OR (95% C.I.)	P value§§
**rs2116830**	All	Additive*	3267/1280/126	3060/1520/192	1.27 (1.13, 1.43)	4.2 ×10^-05^
(*KCNMA1*)		Dominant**	4547/126	4580/192	1.51 (1.20, 1.89)	0.0004
		Recessive§	3267/1406	3060/1712	1.30 (1.19, 1.42)	2.5 × 10^-09^
	Male	Additive	1127/475/48	1311/641/80	1.20 (1.00, 1.44)	0.055
		Dominant	1602/48	1952/80	1.37 (0.95, 1.97)	0.092
		Recessive	1127/523	1311/721	1.19 (1.03, 1.36)	0.016
	Female	Additive	2138/805/78	1747/879/111	1.32 (1.14, 1.53)	0.00024
		Dominant	2943/78	2626/111	1.60 (1.19, 2.14)	0.0019
		Recessive	2138/883	1747/990	1.37 (1.23, 1.53)	2.0 ×10^-08^
**rs988712**	All	Additive	2952/1504/183	2662/1737/327	1.41 (1.28, 1.55)	1.1 ×10^-12^
(*BDNF*)		Dominant	4456/183	4399/327	1.81 (1.50, 2.18)	3.7 × 10^-10^
		Recessive	2952/1687	2662/2064	1.36 (1.25, 1.47)	6.2 × 10^-13^
	Male	Additive	1041/519/82	1106/753/140	1.27 (1.10, 1.46)	0.0011
		Dominant	1560/82	1859/140	1.43 (1.08, 1.90)	0.012
		Recessive	1041/601	1106/893	1.40 (1.22, 1.60)	8.7 × 10^-07^
	Female	Additive	1909/985/101	1554/983/187	1.51 (1.33, 1.71)	1.47 × 10^-10^
		Dominant	2894/101	2537/187	2.11 (1.65, 2.71)	3.4 × 10^-09^
		Recessive	1909/1086	1554/1170	1.32 (1.19, 1.47)	2.4 × 10^-07^

* Numbers of subject with genotype GG, GT or TT, ** Numbers of subject with genotype GG or GT versus number of subjects with genotype TT, and § numbers of subject with genotype GG versus GT and T where G is the effect allele;

§§Model based analysis was carried out by logistic regression, se Statistical analysis.

rs988712*G, which is located 113,058 base pairs downstream of the established obesity locus *BDNF *on chromosome 11 [8], showed consistent effect direction and was significantly associated with obesity in the French cohort 5 (*P *= 0.0020), but not in the German cohort 6 (*P *= 0.135). The overall meta-analysis p-value for association with obesity showed genome-wide significance (*P *= 5.2 × 10^-17^) with an OR of 1.36 [95% C.I. 1.20-1.55] (Figure 2B). There was statistical evidence for heterogeneity in impact on obesity between cohorts for rs988712 (*P *= 0.027). Additive, dominant and recessive genetic models reached genome-wide significance and the G-allele of rs988712 was associated with obesity in both men and women (Table 4). Only two of seven obesity-associated SNPs in the *BDNF *region in [8], rs6265 and rs10501087, were genotyped in our GWA cohort. Neither showed strong LD with rs988712 in our cohort, r2 0.57-0.59.

For the new obesity susceptibility locus rs2116830 (*KCNMA1*) we performed quantitative trait analysis of BMI in three adult population-based samples, n = 4133 in total (Table 1). One of these cohorts (controls in French cohort 5) was also used in the case-control studies described above. For BMI we could detect 1.0 kg/m^2 ^difference between genotypes with 93% power with nominal *P *of 0.001. There was neither significant association with BMI in any population alone, nor in joint analysis of all three populations (additional file 1, Table S3). (The same results were obtained when we included gender, age, and ethnicity in the analysis (results not shown). When we compared allele frequencies between obese cases and non-obese controls in the population based samples we observed consistent effect direction (additional file 1, Table S3) as in the case-control analysis of obesity in cohorts 1-5 described above (Table 2). The results were non-significant; however, this was expected as the total number of obese cases in the population-based samples was small. 48 subjects had BMI > 35 kg/m^2 ^and 267 subjects had BMI > 30 kg/m^2^.

We investigated to what extent our GWA replicated published obesity or BMI loci that had been identified by GWA analysis. For each of the more than 30 published loci, we selected one, or sometimes two, SNPs for which the strongest association with obesity or BMI had been reported. 13 of these SNPs, which represented eleven loci, were analyzed for association with obesity in cohort 1. The remaining SNPs were not represented on the Affymetrix 500K Gene chip arrays used for the GWA or the SNP did not pass our quality control. Besides *BDNF*, our GWA study confirmed allelic association for *MC4R *with nominal *P*< 0.05 (additional file 1, Table S4).

### Adipose tissue expression of KCNMA1 is increased in obesity

**Figure 3:**
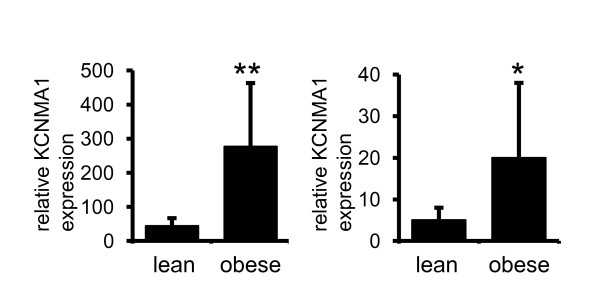
***KCNMA1 *expression in adipose tissue in relation to obesity**. RNA expression in (A) intact adipose tissue and (B) isolated fat cells of lean (n = 5 women, 2 men) and obese (n = 6 women and n = 1 man) subjects. Relative *KCNMA1 *expression = 2^(Ct KCNMA1 calibrator- Ct KCNMA1 sample)^/ 2^(Ct 18S calibrator- Ct 18S sample)^. Two group comparisons were performed with Student's t-test. Values are mean ± SD. *** *P*< 0.0001; * *P*< 0.05

To elucidate potential mechanisms of action of *KCNMA1 *rs2116830 in obesity we determined mRNA levels of in human abdominal subcutaneous adipose tissue from obese and lean subjects. *KCNMA1 *mRNA was increased about fourfold in adipose tissue (*P *= 0.0001) as well as isolated fat cells (*P *= 0.04) derived from obese subjects compared to controls (Figure 3).

## Discussion

We have identified one new susceptibility locus for obesity near *KCNMA1 (*rs2116830) and confirmed association with *BDNF (*rs988712) [8], by GWA analysis in a limited sample of morbidly obese and lean adults This study was followed up by genotyping of five additional European case-control cohorts. Both loci reached genome-wide significant association with obesity in meta-analysis of investigated cohorts [33].

Our exploratory GWA study was performed in morbidly obese subjects raising the question to what extent the results are applicable to other groups of obese patients? Cotsapas et al proposed that severe obesity in most cases is a condition at the extreme of the phenotypic spectrum rather than a distinct condition, which suggests that the same genes are involved in all obese patient groups of the same ethnic origin [6]. However, the susceptibility loci for morbid obesity identified by Meyre et al and Scherag et al seemed to have limited or no impact on BMI in the general population [7,10]. This may also be true for the novel obesity gene *KCNMA1 *identified in this study. *KCNMA1 *rs2116830 was not associated to BMI in three population based samples. It is tempting to speculate that *KCNMA1 *is of minor importance for the development of a moderate increase in fat mass, but contribute to excessive accumulation of adipose tissue in obesity. However, the results from the population-based cohorts should be interpreted with caution as the gender-ratio differed widely between samples. The differences in gender-ratios between the different case-control cohorts were smaller and are thus less likely to confound the results.

Many longitudinal studies show a strong continuity from mild obesity in childhood to more severe obesity in adulthood [34]. In addition, most obesity-susceptibility loci identified by GWA studies in adults are already associated with anthropometric traits in children/adolescents [35]. Together, this supports a shared genetic background for early onset and morbid obesity. There are only few large cohorts available for studies of distinct forms of obesity. In order to obtain genome-wide significance we had to include cohorts with different forms of obesity in the case control analyses [adults with morbid obesity (BMI > 40 kg/m^2^), adult obesity (BMI 30-40 kg/m^2^), and childhood obesity]. The two susceptibility loci for obesity reported here, *KCNMA1 *and *BDNF *displayed nominal allelic association with obesity in each investigated adult case-control cohort. However, the associations of *KCNMA1 *and *BDNF *with childhood obesity are less evident and need to be comfirmed in additional cohorts; with both genes being associated with obesity in French but not in German children. Joint analysis of our GWA data on morbid obese and lean adults, and the GWA data from Hinney's et al on obese children and adult lean controls may help to clarify to what extent morbid and childhood obesity have a similar genetic background [3].

Among obesity and BMI associated loci reported in other GWA studies, we could only confirm two, *BDNF *and *MC4R*. One weakness in our analysis was that we, due to a small sample sizes and different ethnicity, did not perform imputation of all BMI associated loci in published GWAs. Our analysis was therefore limited to published obesity-associated SNPs that produced high quality genotypes in our study, and encompassed only eleven out of more than 30 reported loci. The inability to confirm published obesity loci could be due to small sample size, different ethnicity, differences in sample selection and definition of phenotype. Obesity genes are not necessarily universally detected. Several obesity genes in previous GWAs could not be replicated in large European populations [36,37]. All published obesity loci detected by GWA confer a modest to small risk for obesity. This is also true for *KCNMA1 *and *BDNF *in this study. The odds ratio for developing obesity is 1.26 and 1.36, respectively, for these two loci. The heterogeneity between cohorts for impact of *BDNF *rs988712 on obesity may be due to differences in ethnicity, age, or severity of obesity, as well as low power.

In order to further evaluate mechanisms by which *KCNMA1 *could contribute to the pathogenesis of obesity we performed gene expression studies in adipose tissue and observed increased *KCNMA1 *mRNA expression in obesity. *KCNMA1 *encodes one subunit of the large-conductance voltage- and Ca(2+)-activated K+ channel (BK channel), which is implicated in human epilepsy, blood pressure regulation, and the risk of myocardial infarction [38,39]. In addition, at cellular levels stimulation of *KCNMA1 *channels enhances proliferation of human pre-adipocytes *in vitro *[40]. The latter is intriguing since it has recently been shown that there is a high rate of adipocyte turnover *in vivo*; with about 10% of fat cells being renewed annually [41]. Furthermore, adipocyte number is a major determinant for the fat mass in adults [41]. Thus, *KCNMA1 *could hypothetically contribute to obesity by increasing number of fat cells. We did not have access to tissue samples to study *KCNMA1 *expression in other organs, including the organ strongest implicated in regulation of food intake and obesity, the brain. We therefore cannot exclude a change of *KCNMA1 *expression y in other organs in association with obesity.

Besides small sample size there are other weaknesses in our GWA., e.g. we did not calculate power and ancestry principal components, nor did we formally test for relatedness between subjects.

## Conclusions

In conclusion, we identified *KCNMA1 *as a novel susceptibility locus for obesity, which may promote obesity at least in part by acting in adipose tissue. Furthermore, we confirmed the previously described obesity locus *BDNF*. Further studies of *KCNMA1 *may highlight new targets for treating obesity.

## Competing interests

The authors declare that they have no competing interests.

## Authors' contributions

HJ, PA, ID, KDW and JK designed the study. HJ and DB analyzed the data. PA, JH, BD, SC, FvH, OP, TH, TIAS, JH, AH, and KC collected the samples. PA, ID, TA, JH, JK, and KDW were responsible for genotyping. PA and ID were responsible for mRNA measurements. HJ, PA and ID wrote the manuscript draft. All authors read and approved the final manuscript.

## Pre-publication history

The pre-publication history for this paper can be accessed here:

http://www.biomedcentral.com/1755-8794/4/51/prepub

## Supplementary Material

Additional file 1 ***Supplementary methods, figures and tables***. This file contains additional methodological description of the GWA. It also contains Figure S1 with Q-Q and S2 with Manhattan plots. Finally this file contains Tables S1 showing chromosomal distribution of analyzed 500K SNPs, S2 showing results of replication genotyping, S3 showing association of rs2116830*G with BMI and obesity in population-based samples, and S4 showing replication of published obesity and BMI loci identified by GWA. Click here for file
