# Supplementary material for: Genome wide association study identifies KCNMA1 contributing to human obesity
Source: BMC Med Genomics. 2011 Jun 28;4:51. doi: 10.1186/1755-8794-4-51 (PMC3148553; doi:10.1186/1755-8794-4-51)
Supplement: Additional file 1 — Supplementary methods, figures and tables. This file contains additional methodological description of the GWA. It also contains Figure S1 with Q-Q and S2 with Manhattan plots. Finally this file contains Tables S1 showing chromosomal distribution of analyzed 500K SNPs, S2 showing results of replication genotyping, S3 showing association of rs2116830*G with BMI and obesity in population-based samples, and S4 showing replication of published obesity and BMI loci identified by GWA. [file 1755-8794-4-51-S1.DOC]

**Additional file 1**

### Genome-wide association study

Genome-wide association genotyping

250 ng of genomic DNA from each subject was processed according to the Affymetrix Low throughput protocol for the 500K array set. Labeled probes were hybridized to the Affymetrix 500K array set. Quality control (QC) of the calling results was conducted to remove markers with low quality. Departure from Hardy-Weinberg equilibrium (HWE) was checked for all SNPs using χ^2^ tests. SNPs with a call rate <90%, minor allele frequency (MAF) <0.01, or deviations from HWE *P* <0.001 in controls were excluded from association analysis (Supplementary Table 1).

Follow up of GWA in additional cohorts

The first follow up was performed in Swedish cohort 2. For this analysis we designed two multiplex SNP assays for genotyping with Illumina Golden Gate. We selected SNPs with a high score according to Illumina, thereby validating the use of the designed Illumina genotyping assays. Out of 755 SNPs, 700 fulfilled our quality criteria. The mean call rate for these SNPs was 99.1% (range 90.3-99.6%). In all, 35 SNPs were excluded due to <90% call rate and 20 SNPs due to HWE *P*<0.01. For genotyped SNPs, there was 99.98% concordance between duplicate control samples. The 23 SNPs nominally associated with obesity in cohort 2 were all in HWE.

**Supplementary figures**

**Figure S1.** **Q-Q plot of genome-wide allelic association analysis of obesity for chromosomes 1 to 22.** P values were presented in a log 10-transformation format.

**Figure S2. Manhattan plot of genome-wide allelic association analysis of obesity for chromosomes 1 to 22.** P values were presented in a log 10-transformation format and real chromosomal positions were used.

**Table S1. Chromosomal distribution of SNPs from 500K arrays (Sty and Nsp) and genotype quality control**

| **Chr** | **Number**  **of SNPs** | | **Call rate (%)** | | **Number of SNPs with call rate <90%** | | **Number of SNPs with MAF <1%** | | **Number of SNPs with HWE *P*<0.001** | | **SNPs analyzed**  **in obesity** | |
| --- | --- | --- | --- | --- | --- | --- | --- | --- | --- | --- | --- | --- |
|  | Sty | Nsp | Sty | Nsp | Sty | Nsp | Sty | Nsp | Sty | Nsp | Sty | Nsp |
| 1 | 20338 | 19865 | 98.7 | 98.4 | 490 | 601 | 3294 | 3057 | 209 | 217 | 16415 | 16077 |
| 2 | 19182 | 22213 | 98.7 | 98.4 | 450 | 716 | 2830 | 3106 | 174 | 220 | 15782 | 18257 |
| 3 | 15413 | 18379 | 98.8 | 98.4 | 367 | 574 | 2189 | 2444 | 161 | 208 | 12749 | 15223 |
| 4 | 13258 | 19058 | 98.7 | 98.4 | 319 | 557 | 1962 | 2824 | 130 | 188 | 10887 | 15558 |
| 5 | 14889 | 17164 | 98.7 | 98.4 | 324 | 553 | 1917 | 2125 | 139 | 167 | 12548 | 14386 |
| 6 | 14322 | 17144 | 98.7 | 98.4 | 335 | 529 | 1616 | 1869 | 169 | 181 | 12244 | 14622 |
| 7 | 11880 | 13950 | 98.7 | 98.4 | 291 | 437 | 1410 | 1632 | 139 | 171 | 10082 | 11770 |
| 8 | 12615 | 14839 | 98.7 | 98.5 | 315 | 418 | 1791 | 1935 | 109 | 161 | 10436 | 12379 |
| 9 | 10918 | 11941 | 98.7 | 98.4 | 268 | 346 | 1364 | 1429 | 101 | 127 | 9214 | 10089 |
| 10 | 14220 | 14277 | 98.7 | 98.4 | 350 | 411 | 1933 | 1826 | 138 | 136 | 11840 | 11948 |
| 11 | 12963 | 13306 | 98.8 | 98.3 | 299 | 425 | 1894 | 1793 | 124 | 160 | 10685 | 10985 |
| 12 | 11890 | 13058 | 98.7 | 98.4 | 296 | 409 | 1587 | 1719 | 127 | 159 | 9921 | 10836 |
| 13 | 8067 | 11117 | 98.7 | 98.4 | 195 | 316 | 1129 | 1527 | 88 | 105 | 6680 | 9202 |
| 14 | 7536 | 8181 | 98.7 | 98.3 | 164 | 259 | 1075 | 1120 | 72 | 91 | 6252 | 6747 |
| 15 | 7338 | 7011 | 98.7 | 98.4 | 172 | 254 | 1133 | 1032 | 81 | 94 | 5975 | 5669 |
| 16 | 8285 | 7024 | 98.8 | 98.5 | 179 | 209 | 1344 | 1008 | 83 | 87 | 6707 | 5755 |
| 17 | 6423 | 4854 | 98.8 | 98.4 | 148 | 157 | 895 | 659 | 66 | 76 | 5327 | 3989 |
| 18 | 6732 | 8148 | 98.8 | 98.4 | 150 | 233 | 1012 | 1204 | 58 | 69 | 5534 | 6669 |
| 19 | 3704 | 2692 | 98.8 | 98.3 | 88 | 107 | 479 | 350 | 48 | 38 | 3101 | 2212 |
| 20 | 6561 | 5837 | 98.8 | 98.5 | 133 | 171 | 906 | 766 | 62 | 68 | 5482 | 4866 |
| 21 | 3189 | 3935 | 98.7 | 98.4 | 74 | 124 | 395 | 554 | 42 | 41 | 2694 | 3233 |
| 22 | 3687 | 2519 | 98.8 | 98.5 | 81 | 83 | 509 | 320 | 50 | 38 | 3060 | 2090 |
| **total** | **233410** | **256512** | **98.7** | **98.4** | **5488** | **7889** | **32664** | **34299** | **2370** | **2802** | **193615** | **212562** |

Chr=chromosome, MAF=minor allele frequency

**Table S2. Replication of obesity associationsb, e**

| **cohort** |  | **1 (Obese n=164, Lean n=163)^c^** | | | **2 (Obese n=460, Lean n=247)^c^** | | |  |
| --- | --- | --- | --- | --- | --- | --- | --- | --- |
| **SNPs** | **allele** | **Obese %** | **Lean %** | ***P*** | **Obese %** | **Lean %** | ***P*** |  |
| **rs871343** | G | 68 | 56 | 0.0023 | 64 | 58 | 0.027 |  |
| **rs2781162** | G | 69 | 57 | 0.0026 | 62 | 56 | 0.037 |  |
| **rs12120304** | A | 56 | 43 | 0.0025 | 51 | 42 | 0.0012 |  |
| **rs1533948** | T | 73 | 62 | 0.0021 | 65 | 60 | 0.043 |  |
| **rs4552318** | C | 86 | 76 | 0.0019 | 81 | 77 | 0.039 |  |
| **rs869572** | T | 85 | 75 | 0.0022 | 80 | 76 | 0.097 |  |
| **rs17116175** | G | 94 | 85 | 0.00017 | 90 | 87 | 0.04 |  |
| **rs1452835** | T | 76 | 63 | 0.0006 | 77 | 68 | 0.0004 |  |
| **rs16900858** | C | 8 | 3 | 0.0063 | 7 | 4 | 0.023 |  |
| **rs12698956** | C | 91 | 81 | 0.00063 | 92 | 88 | 0.013 |  |
| **rs7012413** | T | 34 | 23 | 0.0017 | 31 | 24 | 0.0064 |  |
| **rs2165414** | A | 75 | 64 | 0.0023 | 70 | 65 | 0.028 |  |
| **rs10118276** | C | 63 | 51 | 0.0028 | 58 | 49 | 0.0028 |  |
| **rs10970653** | G | 93 | 86 | 0.0044 | 91 | 86 | 0.018 |  |
| **rs2116830** | G | 87 | 76 | 0.0005 | 84 | 79 | 0.019 |  |
| **rs621314** | C | 38 | 25 | 0.00082 | 34 | 29 | 0.035 |  |
| **rs988712** | G | 81 | 73 | 0.0017 | 82 | 76 | 0.0095 |  |
| **rs10783807** | C | 59 | 46 | 0.001 | 55 | 48 | 0.015 |  |
| **rs7310454** | T | 54 | 42 | 0.0042 | 48 | 41 | 0.014 |  |
| **rs3109610** | C | 99 | 96 | 0.0029 | 95 | 92 | 0.046 |  |
| **rs4464125** | A | 28 | 17 | 0.00056 | 28 | 20 | 0.002 |  |
| **rs12969426** | G | 93 | 86 | 0.0025 | 90 | 87 | 0.031 |  |
| **rs1260849^a^** | G | 75 | 64 | 0.0025 | 70 | 63 | 0.006 |  |
|  |  |  |  |  |  |  |  |  |
| **cohort** |  | **3 (Ob N=1814, Le N=1704)^c^** | | | **4 (Ob N=985, Le N=532)^c^** | | | **Pooled** |
| **SNPs** | **allele** | **Obese %** | **Lean %** | ***P*** | **Obese %** | **Lean %** | ***P*** | ***P*** |
| **rs871343** | G | 59 | 59 | 0.87 | 51 | 61 | 1.0x10^-6^ | 0.035 |
| **rs2781162** | G | 59 | 59 | 0.7 | 52 | 59 | 0.001 | 0.089 |
| **rs12120304** | A | 49 | 47 | 0.19 | 39 | 49 | 5x10^-8^ | 0.67 |
| **rs1533948** | T | 66 | 67 | 0.52 | 63 | 61 | 0.32 | 0.42 |
| **rs4552318** | C | 79 | 79 | 0.76 | 0.76 | 0.72 | 0.014 | 0.35 |
| **rs869572** | T | 21 | 21 | 0.83 | 0.25 | 0.2 | 0.0014 | 0.66 |
| **rs17116175** | G | 89 | 90 | 0.81 | 92 | 91 | 0.16 | 0.012 |
| **rs1452835** | T | 73 | 71 | 0.075 | 0.71 | 0.7 | 0.79 | 0.0092 |
| **rs16900858** | C | 94 | 94 | 0.88 | 0.96 | 0.95 | 0.18 | 0.53 |
| **rs12698956^d^** | C | 90 | 91 | 0.2 |  |  |  |  |
| **rs7012413** | T | 30 | 29 | 0.19 | 38 | 33 | 0.0023 | 2.8x10^-6^ |
| **rs2165414** | A | 70 | 72 | 0.1 | 74 | 71 | 0.065 | 0.55 |
| **rs10118276** | C | 53 | 53 | 0.86 | 56 | 52 | 0.06 | 0.004 |
| **rs10970653** | G | 9 | 10 | 0.49 | 0.09 | 0.09 | 0.59 | 0.047 |
| **rs2116830** | G | 83 | 81 | 0.024 | 0.85 | 0.82 | 0.024 | 9.4 x10^-6^ |
| **rs621314** | C | 33 | 33 | 0.82 | 38 | 35 | 0.06 | 0.01 |
| **rs988712** | G | 80 | 76 | 4.60E-05 | 0.81 | 0.71 | 2.2 x10^-9^ | 1.4 x10^-13^ |
| **rs10783807** | C | 53 | 52 | 0.18 | 0.51 | 0.49 | 0.29 | 0.19 |
| **rs7310454** | T | 45 | 45 | 0.87 | 0.45 | 0.42 | 0.14 | 0.013 |
| **rs3109610** | C | 93 | 93 | 0.97 | 92 | 92 | 0.68 | 0.32 |
| **rs4464125** | A | 25 | 24 | 0.4 | 26 | 24 | 0.29 | 0.0052 |
| **rs12969426** | G | 89 | 89 | 0.57 | 0.93 | 0.9 | 0.0039 | 0.0003 |
| **rs1260849^a^** | G | failed |  |  | 65 | 62 | 0.1 | 0.92 |

1. This SNP is in close LD with rs10402812 that was genotyped in cohorts 2-3. b) All SNPs in cohorts 1 and 2 are in HWE with *P*>0.05. In cohorts 3 rs10402812 is not in HWE, whereas remaining SNPs have HWE *P*>0.01; c) Frequency of risk allele among Obese, and controls; d) We could not design a Sequenom genotyping assay for this SNP and we had no nearby SNP that was associated with obesity that could be genotyped. e) Genotype call rates are in cohort 1 mean 98% (range 91-99), cohort 2 mean 95% (range 83-100 ), cohort 3 mean 96% (range 84-99), and cohort 4 mean 95% (range 87-99). Ch=chromosome, call=genotype call rate

**Table S3. Association of rs2116830*G with BMI and obesity in population-based samples**

|  | **Quantitative analysis of BMI**^c^ | | | | | | | | | **Qualitative analysis of obesity**^d^ | | | | | | |  |
| --- | --- | --- | --- | --- | --- | --- | --- | --- | --- | --- | --- | --- | --- | --- | --- | --- | --- |
| **cohort** | **n^a^** | **mean±SD^b^** | **n** | **mean±SD** | **n** | **mean±SD** | **BETA** | **SE** | ***P*** | **obese** | **nonobese** | | | **OR (95% C.I.)** | ***P*** | | |
|  | **G/G** | | **G/T** | | **T/T** | |  | | |  | | | | | | |  |
| French 5 | 1010 | 23.8±3.6 | 555 | 24.0±3.6 | 74 | 23.3±3.4 | -0.008 | 0.15 | 0.96 | 79 | | 78 | 1.05 (0.70,1.56) | | | 0.83 |  |
| Swedes 8 | 484 | 24.8±4.3 | 248 | 24.4±3.8 | 28 | 24.1±3.4 | 0.34 | 0.26 | 0.20 | 81 | | 79 | 1.15 (0.74,1.77) | | | 0.53 |  |
| Danish 9 | 572 | 26.0±3.6 | 249 | 26.3±3.4 | 26 | 25.5±5.1 | -0.09 | 0.23 | 0.68 | 82 | | 82 | 0.97 (0.66,1.44) | | | 0.89 |  |
| Pooled | 2066 | 24.6±3.9 | 1052 | 24.6±3.7 | 128 | 24.0±3.9 | 0.14 | 0.12 | 0.23 | 81 | | 79 | 1.10 (0.88,1.39) | | | 0.4 |  |

a) n=number of subjects with indicated genotype; b) mean**±**SD of BMI (kg/m^2^) in each genotype group; c) Quantitative analysis of BMI was performed with linear regression, see Statistical analysis. d) Obesity is defined as BMI >35 kg/m^2^. Risk allele frequencies were compared between obese cases and controls by Chi^2^-test.

| **Table S4 Replication of published obesity and BMI loci identified by GWA** | | | | | | |  | | |
| --- | --- | --- | --- | --- | --- | --- | --- | --- | --- |
| **Chr** | **Nearby gene** | **SNP** | **Risk allele^a^** | **Obese %^b^** | **Lean %^b^** | **OR (95% C.I.)** | | ***P***^c^ | **Reference** |
| 1 | *LYPLAL1* | rs2605100 | G | 33.8 | 30.4 | 1.17(0.84,1.63) | | 0.34 | (1) |
| 1 | *TNNI3K* | rs1514175 | A | 49.7 | 44.2 | 1.25(0.92,1.70) | | 0.16 | (2) |
| 1 | *PTBP2* | rs1555543 | C | 64.0 | 57.4 | 1.32(0.97,1.81) | | 0.08 | (2) |
| 2 | *INSIG2* | rs7566605 | C | 34.8 | 34 | 1.03(0.75,1.42) | | 0.85 | (3) |
| 2 | *TMEM18* | rs6548238 | C | 19.5 | 13.9 | 1.5(0.98,2.29) | | 0.059 | (4) |
| 11 | *BDNF* | rs10501087 | T | 24.4 | 16.2 | 1.67(1.14,2.46) | | 0.009 | (5) |
| 11 | *BDNF* | rs6265 | G | 23.8 | 15.8 | 1.66(1.12,2.45) | | 0.01 | (5) |
| 12 | *BCDIN3D-FAIM2* | rs7138803 | A | 41.2 | 40.9 | 1.01(0.74,1.39) | | 0.94 | (5) |
| 16 | *FTO* | rs1121980 | T | 47 | 42.6 | 1.19(0.87,1.62) | | 0.27 | (6) |
| 16 | *FTO* | rs9939609 | A | 45.7 | 40.2 | 1.25(0.92,1.70) | | 0.16 | (6) |
| 16 | *RPGRIP1L* | rs8050136 | A | 45.7 | 40.5 | 1.24(0.91,1.69) | | 0.18 | (5) |
| 18 | *MC4R* | rs17700633 | T | 37.2 | 29.9 | 1.39(1.01,1.92) | | 0.047 | (7) |
| 20 | *CTNNBL1* | rs6013029 | G | 5.6 | 5.5 | 1.01(0.52,1.98) | | 0.97 | (8) |
| a) Allele associated with higher BMI or obesity in the literature. b) Frequency of published risk allele in our obese cases (n=164) and lean controls (n=163). c) Risk allele frequencies were compared between obese cases and controls by Chi^2^-test. Chr=chromosome | | | | | | | | | |


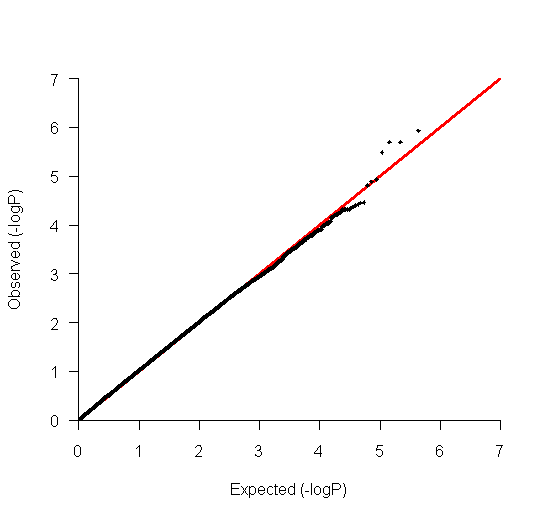
**Figure S1**

**Figure S2**

**
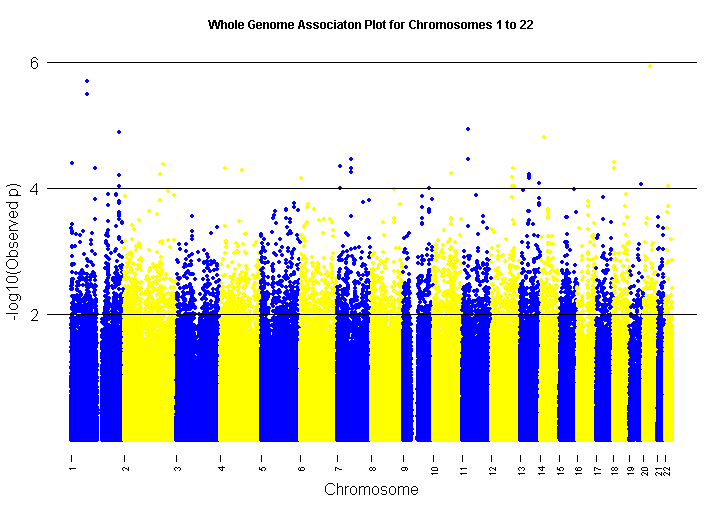
**

**References**

**9 Speliotes E. K. et al. (2010) Association analyses of 249,796 individuals reveal 18 new loci associated with body mass index.** [Nat Genet.](javascript:AL_get(this, 'jour', 'Nat Genet.');) Nov;42(11):937-48

**1 Lindgren, C.M., Heid, I.M., Randall, J.C., Lamina, C., Steinthorsdottir, V., Qi, L., Speliotes, E.K., Thorleifsson, G., Willer, C.J., Herrera, B.M. *et al.* (2009) Genome-wide association scan meta-analysis identifies three Loci influencing adiposity and fat distribution. *PLoS Genet*, 5, e1000508.**

**2 Speliotes, E.K., Willer, C.J., Berndt, S.I., Monda, K.L., Thorleifsson, G., Jackson, A.U., Allen, H.L., Lindgren, C.M., Luan, J., Magi, R. *et al.* Association analyses of 249,796 individuals reveal 18 new loci associated with body mass index. *Nat Genet*, 42, 937-948.**

**3 Heid, I.M., Huth, C., Loos, R.J., Kronenberg, F., Adamkova, V., Anand, S.S., Ardlie, K., Biebermann, H., Bjerregaard, P., Boeing, H. *et al.* (2009) Meta-analysis of the INSIG2 association with obesity including 74,345 individuals: does heterogeneity of estimates relate to study design? *PLoS Genet*, 5, e1000694.**

**4 Willer, C.J., Speliotes, E.K., Loos, R.J., Li, S., Lindgren, C.M., Heid, I.M., Berndt, S.I., Elliott, A.L., Jackson, A.U., Lamina, C. *et al.* (2009) Six new loci associated with body mass index highlight a neuronal influence on body weight regulation. *Nat Genet*, 41, 25-34.**

**5 Thorleifsson, G., Walters, G.B., Gudbjartsson, D.F., Steinthorsdottir, V., Sulem, P., Helgadottir, A., Styrkarsdottir, U., Gretarsdottir, S., Thorlacius, S., Jonsdottir, I. *et al.* (2009) Genome-wide association yields new sequence variants at seven loci that associate with measures of obesity. *Nat Genet*, 41, 18-24.**

**6 Frayling, T.M., Timpson, N.J., Weedon, M.N., Zeggini, E., Freathy, R.M., Lindgren, C.M., Perry, J.R., Elliott, K.S., Lango, H., Rayner, N.W. *et al.* (2007) A common variant in the FTO gene is associated with body mass index and predisposes to childhood and adult obesity. *Science*, 316, 889-894.**

**7 Loos, R.J., Lindgren, C.M., Li, S., Wheeler, E., Zhao, J.H., Prokopenko, I., Inouye, M., Freathy, R.M., Attwood, A.P., Beckmann, J.S. *et al.* (2008) Common variants near MC4R are associated with fat mass, weight and risk of obesity. *Nat Genet*, 40, 768-775.**

**8 Liu, Y.J., Liu, X.G., Wang, L., Dina, C., Yan, H., Liu, J.F., Levy, S., Papasian, C.J., Drees, B.M., Hamilton, J.J. *et al.* (2008) Genome-wide association scans identified CTNNBL1 as a novel gene for obesity. *Hum Mol Genet*, 17, 1803-1813.**
